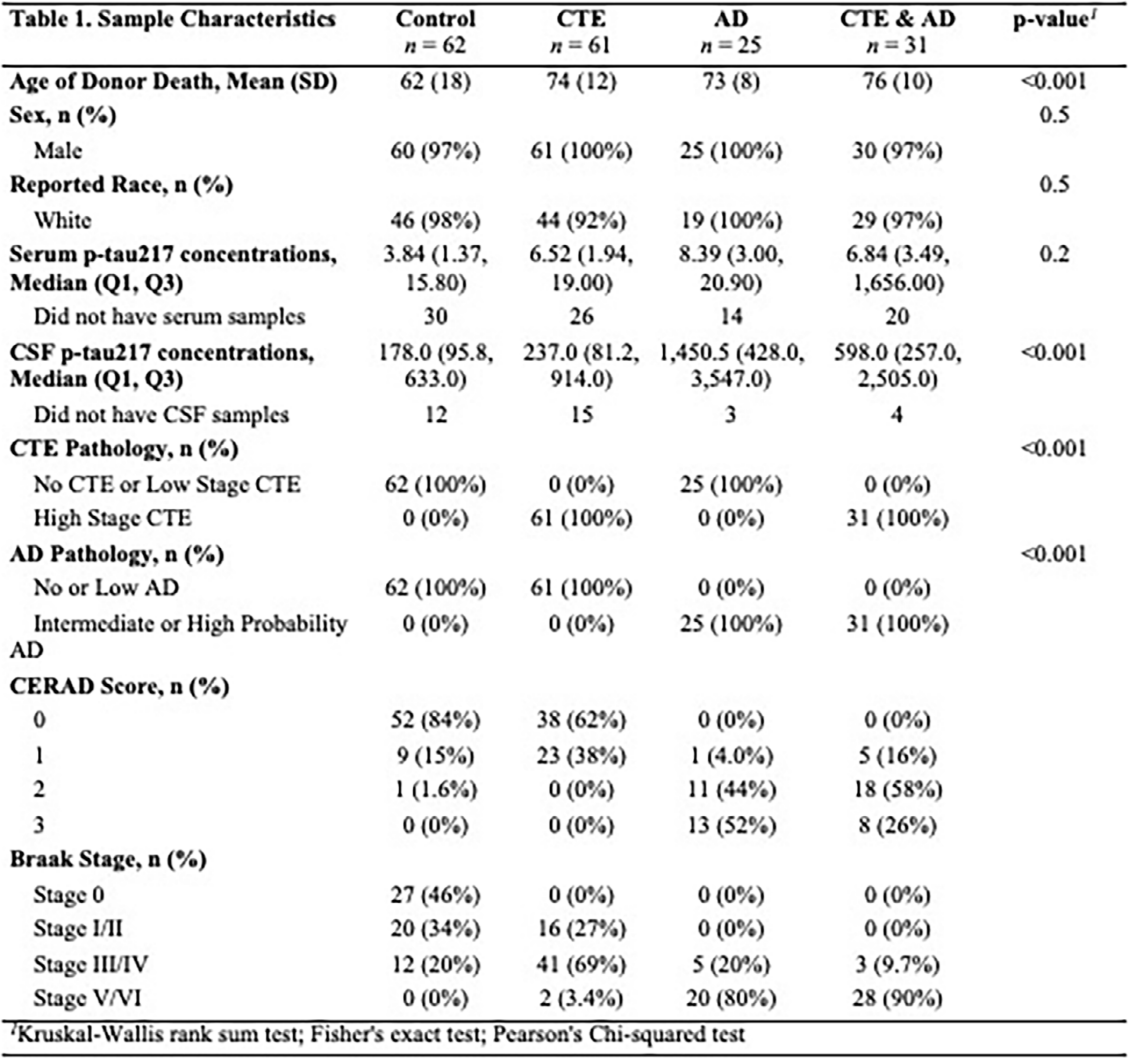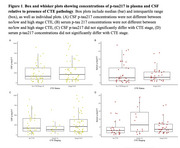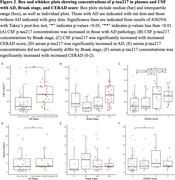# The utility of phosphorylated Tau‐217 in discriminating chronic traumatic encephalopathy and Alzheimer’s disease in postmortem cerebral spinal fluid and serum

**DOI:** 10.1002/alz70861_108171

**Published:** 2025-12-23

**Authors:** William S Cole‐French, Petra Ypsilantis, Guglielmo Di Molfetta, Jenna R. Groh, Annalise E. Miner, Anna Aaronson, Raymond Nicks, Elizabeth Spurlock, Sophia Nosek, Samantha I Hawkins, Shania C. Mulayi, Kelsey Goostrey, Matthew Roebuck, Kerry Cormier, Renecca Mathias, Caroline Kubilus, Ann C. McKee, Yorghos Tripodis, Laia Montoliu‐Gaya, Jesse Mez, Nicholas J. Ashton, Michael L Alosco, Thor D. Stein

**Affiliations:** ^1^ Boston University Chronic Traumatic Encephalopathy, Chobanian & Avedisian School of Medicine, Boston, MA USA; ^2^ Department of Psychiatry and Neurochemistry, Institute of Neuroscience and Physiology, The Sahlgrenska Academy, University of Gothenburg, Mölndal Sweden; ^3^ Institute of Neuroscience and Physiology, University of Gothenburg, Mölndal Sweden; ^4^ Boston University Alzheimer’s Disease Research and CTE Centers, Boston University Chobanian & Avedisian School of Medicine, Boston, MA USA; ^5^ Boston University Alzheimer’s Disease Research Center, Boston, MA USA; ^6^ Boston University CTE and Alzheimer's Disease Research Center, Boston, MA USA; ^7^ Boston University Chronic Traumatic Encephalopathy Chobanian and Avedisian School of Medicine, Boston, MA USA; ^8^ Boston University Chobanian & Avedisian School of Medicine, Boston, MA USA; ^9^ Boston University Chronic Traumatic Encephalopathy Center, Boston University Chobanian & Avedisian School of Medicine, Boston, MA USA; ^10^ Boston University Chronic Traumatic Encephalopathy Center, Boston, MA USA; ^11^ Boston University School of Public Health, Boston, MA USA; ^12^ Department of Psychiatry and Neurochemistry, Institute of Neuroscience and Physiology, The Sahlgrenska Academy, University of Gothenburg, Mölndal, Gothenburg Sweden; ^13^ Alzheimer’s Disease Research Center, Boston University Chobanian & Avedisian School of Medicine, Boston, MA, USA, Boston, MA USA; ^14^ Banner Alzheimer's Institute, Phoenix, AZ USA; ^15^ Department of Psychiatry and Neurochemistry, Institute of Neuroscience & Physiology, the Sahlgrenska Academy at the University of Gothenburg, Mölndal Sweden; ^16^ Institute of Neuroscience and Physiology, Sahlgrenska Academy at the University of Gothenburg, Gothenburg Sweden

## Abstract

**Background:**

Phosphorylated tau (*p* ‐tau)217 has emerged as the optimal blood‐based biomarker for assessing Alzheimer’s disease (AD) pathology *in vivo*. However, the ability of *p* ‐tau217 to detect other neurodegenerative diseases such as chronic traumatic encephalopathy (CTE) is unknown.

**Methods:**

The sample included UNITE brain bank donors with available CSF(*n*=145) and serum (*n*=89) collected postmortem. Neuropathological assessments were performed for CTE, including stages I‐IV, as well as intermediate to high likelihood of AD with NIA‐Reagan criteria, including Braak neurofibrillary tangle stage and CERAD score for neuritic plaques. CTE & AD co‐pathologies were assessed. A commercially available single molecule assay for *p* ‐tau217 (AlzPath) analyzed concentrations within CSF and serum. One‐way ANOVAs were performed to test for increased *p* ‐tau217 levels in CTE and AD compared to controls. Secondary binary and ordinal logistic regressions, adjusting for age, were conducted to test associations of CSF and serum *p* ‐tau217 concentrations with high stage CTE and CTE stage and AD, Braak stage, and CERAD score.

**Results:**

Table 1 describes sample characteristics. *p* ‐tau217 concentrations in CSF and serum show no significant difference in high stage CTE compared to no or low stage CTE or between CTE stages (Figure 1). *p* ‐tau217 in CSF and serum was significantly greater in AD (*p* =<0.001, *p* =0.042) compared to no or low AD and significantly increased in those with CERAD score>1 compared to those with CERAD of 0 (*p* =<0.001, *p* =0.044, Figure 2). Logistic regressions showed no significant associations in CSF or serum between *p* ‐tau217 and CTE or CTE stage. However, CSF *p* ‐tau217 concentrations were positively associated with AD (OR=2.717, *p* =0.002), increasing Braak stage (OR=1.728, *p* =0.004), and with increasing CERAD score (OR=2.048, *p* =<.001). Increasing serum *p* ‐tau217 concentrations showed a trend towards an association with AD (OR=1.456, *p* =0.092) and with increasing CERAD score (OR=1.412, *p* =0.080).

**Conclusions:**

Postmortem CSF and serum *p* ‐tau217 were significantly associated with AD. In contrast, neither CSF or serum *p* ‐tau217 showed associations with CTE diagnosis or severity. This suggests that *p* ‐tau217 may be helpful for discriminating AD from CTE. Discovery efforts are needed for development of fluid biomarkers with diagnostic utility for CTE.